# Online Health Information–Seeking Behaviors Among the Chongqing Population: Cross-Sectional Questionnaire Study

**DOI:** 10.2196/56028

**Published:** 2025-05-05

**Authors:** Honghui Rong, Lu Lu, Miao He, Tian Guo, Xian Li, Qingliu Tao, Yixin Li, Chuanfen Zheng, Ling Zhang, Fengju Li, Dali Yi, Enyu Lei, Ting Luo, Qinghua Yang, Ji-an Chen

**Affiliations:** 1 Department of Health Education, School of Military Preventive Medicine, Army Medical University (Third Military Medical University), Gaotanyan Street No 30, Shapingba District, Chongqing, 400038, China, 86 02368771579; 2 Chongqing Health Education Institute, Chongqing, China

**Keywords:** online health information seeking, health behavior, Chongqing, China, Internet

## Abstract

**Background:**

With the rapid development of the internet and its widespread use, online health information–seeking (OHIS) has become a popular and important research topic. Various benefits of OHIS are well recognized. However, OHIS seems to be a mixed blessing. Research on OHIS has been reported in Western countries and in high-income regions in eastern China. Studies on the population in the western region of China, such as Chongqing, are still limited.

**Objective:**

The aim of the study was to identify the prevalence, common topics, and common methods of health information–seeking and the factors influencing these behaviors among the Chongqing population.

**Methods:**

This cross-sectional questionnaire study was conducted from September to October 2021. A web-based questionnaire was sent to users aged 15 years and older in Chongqing using a Chinese web-based survey hosting site (N=14,466). Data on demographics, web-based health information resources, and health topics were collected. Factors that may influence health literacy were assessed using the chi-square test and multivariate logistic regression models.

**Results:**

A total of 67.1% (9704/14,466) of the participants displayed OHIS behaviors. Participants who were younger, had a higher educational level, and worked as medical staff or teachers were more likely to engage in OHIS, while those living in rural areas, ethnic minorities, and farmers were less likely to seek health information on the web (*P*<.01). Among the Chongqing population, the most common topic searched on the internet was health behavior and literacy (87.4%, 8483/9704), and the most popular method of seeking health information on the web was through WeChat (77.0%, 7468/9704).

**Conclusions:**

OHIS is prevalent in Chongqing. Further research could be performed based on the influencing factors identified herein and high-priority, effective ways of improving the OHIS behaviors of the Chongqing population.

## Introduction

With rapid development, the internet has become a major source of health information worldwide. According to Internet World Stats [1], there are 5 billion internet users, accounting for more than 60% of the world population. China also has a large population of internet users. In 2020, approximately 9.89 million Chinese people (70.4% of the Chinese population) had access to the internet [2]. The internet hosts a tremendous amount and variety of health-related information that can be accessed at conveniently, anonymously, and at relatively low cost [3,4]. Among all health information sources, the internet has the highest usage rate [5-7]. Although certain groups still rely on traditional sources such as books and printed journals for health information [8], web-based sources of health information are increasingly growing in popularity.

Various benefits of online health information–seeking (OHIS) are well recognized. Patients turning to the internet before seeking medical consultations or diagnosis may help improve patient-doctor relationships, and patients are more inclined to trust their physicians’ advice when they are able to discuss the information they found on the web with their doctors [9-12]. As individuals aim to change their lifestyle or health behavior, the frequency of the use of the internet to retrieve health information is likely to increase [13]. For people with chronic diseases, OHIS may help them manage their health condition [14,15]. However, OHIS seems to be a mixed blessing. Studies have reported that the overall quality of web-based health information is relatively low [16-18]. Information seekers are at risk of making hasty or dangerous health decisions based on questionable web-based information [19]. In contrast to the information available in Western countries, low-quality web-based health information seems to be particularly prevalent in Asian countries [20].

With the rapid development of the internet and its widespread use, OHIS has become a popular and important research topic. Research on OHIS has been reported in Western countries [8,14,21] as well as low- and middle-income countries [22-24]. Various factors, such as age, gender, education, and internet usage, have been shown to affect the prevalence and extent of OHIS in previous studies [13,22,25]. In addition, researchers have been interested in the topic of OHIS. A previous study also reported that various health topics are searched by web-based health information seekers. The most common topics searched on the internet are likely to fall under 2 categories: health behavior, such as nutrition or diet, exercise, and body maintenance; and medical concerns, including information related to disease, medications, and treatments [11,14,22,26]. In recent years, several academic studies on OHIS have collected evidence from high-income regions in eastern China, such as Zhejiang, Guangdong, and Hong Kong [26-30]. Western China, as a transitional region—home to 27% of the national population with distinct socioeconomic and health care system characteristics—remains critically underresearched [31]. This knowledge gap limits our understanding of the situation of OHIS thoroughly in this vast country with uneven development. Chongqing, as the largest municipality in western China, embodies the region’s characteristic “urban-rural dual structure” with more than a 60% urbanization rate and significant health resource disparities between metropolitan and rural areas [32]. This metropolis is an ideal context for understanding situation of OHIS in western China.

The study objectives were to (1) determine the prevalence, common topics, and methods of OHIS in the Chongqing population and (2) identify the factors that influence the OHIS behaviors of this population. This research may help improve ways of promoting efficient and appropriate OHIS for users and harnessing the benefits of the internet as a source of health information.

## Methods

### Study Design

From September to October 2021, Chongqing Health Education Institution and the Army Medical University carried out a web-based study to assess residents’ health care needs in Chongqing municipality. The target participants were Chongqing residents. A survey QR code was disseminated on popular Chinese social media applications such as WeChat for voluntary participation in our web-based survey, which was hosted on the Chinese Sojump site. The first page of the survey was a web-based consent form including study information. After reading the consent forms and indicating consent to participate in the survey, respondents were allowed to proceed. To avoid multiple submissions, only one submission per IP address was allowed. Ultimately, 14,466 participants were included in this study.

### Measures

This questionnaire included 2 parts: demographic characteristics and OHIS behaviors. Demographic characteristics included age, gender, education, occupation, ethnicity, and area of residence, gender, education, and ethnicity. Age was measured by asking participants to indicate their numeric age, and other variables were measured with multiple-choice questions. The part on OHIS behaviors were included three questions. The first one dealt addressed having experience with OHIS, wherein participants’ OHIS behavior was measured with a single question, “Which sources are your main sources for seeking health information?” The response options included books and journals, broadcasts, television, PCs (desktops and laptops), mobile phones, lectures, professional staff, and advertisements. Participants who chose PCs and/or mobile phones as their main sources for seeking health information were considered to have experience with OHIS. The second one addressed major health topics in OHIS: health topics that the participants searched on the internet were captured by a multiple-choice question with response options including health behavior and literacy (such as diet, fitness, exercise, and drug usage), infectious diseases, chronic disease, first aid, and health policies (such as medical insurance). Participants could select one or more answers as their major health topics when searching on the internet. The third one addressed the main method of OHIS: the main method of seeking health information on the web was measured with a single item with response options including WeChat, Weibo, search engines (such as Baidu and Google), websites, short-video apps (such as TikTok), and others. Participants could select one or more answers as their main method of seeking health information on the web.

### Ethical Considerations

The study was carried out in accordance with ethical principals and was approved by the ethics review board of the Army Medical University (2023-5-02). Participants provided informed consent. All participants’ information was anonymized. There was no financial compensation for patients or researchers nor any source of funding that could lead to a conflict of interest for the study.

### Statistical Analysis

All data were input into an Epidata database (version 3.1) after checking and correcting errors. SPSS (version 22.0; IBM Corp) was used for analyses. A descriptive analysis (frequencies, percentages, and means with SDs) of the participant characteristics was performed. The chi-square test was used to compare OHIS behaviors among groups. Multiple logistic regression models were used to assess the influencing factors associated with OHIS. Statistical significance was set to a *P* value of <.05 (2-sided).

## Results

### Sociodemographic Characteristics of the Study Sample

The demographic characteristics of the study sample are listed in Table 1. In total, 67.1% (9704/14,466) of the participants had OHIS experience. The average age was 46.2 (SD 18.0) years, while approximately half (51.8%, 7495/14,466) of the participants were younger than 45 years. More than half (52.0%, 7520/14,466) of the participants were female. Most participants (95.3%, 13,793/14,466) were of Han Chinese ethnicity. The percentage of participants residing in urban areas was 69.7% (10,090/14,466). Overall, 47.1% (6813/14,466) of participants were college graduates or had a higher level of education. The participants spanned all occupation groups.

As indicated in Table 1, OHIS experience significantly differed by age, ethnicity, area of residence, education and occupation (*P*<.05) but not by gender.

**Table 1.** Differences in the OHIS^a^ characteristics of the respondents and their sociodemographic characteristics (N=14,466).

Characteristics	Participants, n (%)	Respondents without OHIS experience, n (%)	Respondents with OHIS experience, n (%)	*P* value
**Age (years)**				
15‐45	7495 (51.8)	1208 (25.4)	6287 (64.8)	<.001
46‐60	3036 (21.0)	790 (16.6)	2246 (23.1)	
61 or older	3935 (27.2)	2764 (58.0)	1171 (12.1)	
**Gender**				
Male	6946 (48.0)	2308 (48.5)	4638 (47.8)	.46
Female	7520 (52.0)	2454 (51.5)	5066 (52.2)	
**Ethnicity**				
Han Chinese	13,793 (95.3)	4513 (94.8)	9280 (95.6)	.02
Ethnic minority	673 (4.7)	249 (5.2)	424 (4.4)	
**Area of residence**				
Urban	10,090 (69.7)	2587 (54.3)	7503 (77.3)	<.001
Rural	4376 (30.3)	2175 (45.7)	2201 (22.7)	
**Education level**				
Primary school or less	2998 (20.7)	2301 (48.3)	697 (7.2)	<.001
Junior high school	2467 (17.1)	1020 (21.4)	1447 (14.9)	
Senior high school	2188 (15.1)	514 (10.8)	1674 (17.3)	
College graduate	6289 (43.5)	855 (18.0)	5434 (56.0)	
Postgraduate	524 (3.6)	72 (1.5)	452 (4.7)	
**Occupation**				
Civil servants	1669 (11.5)	253 (5.3)	1416 (14.6)	<.001
Teachers	1208 (8.4)	150 (3.1)	1058 (10.9)	
Medical staff	1603 (11.1)	223 (4.7)	1380 (14.2)	
Staff in public institutions	969 (6.7)	175 (3.7)	794 (8.2)	
Students	887 (6.1)	171 (3.6)	716 (7.4)	
Farmers	3317 (22.9)	2266 (47.6)	1051 (10.8)	
Workers	1180 (8.2)	407 (8.5)	773 (8.0)	
Enterprise personnel	1250 (8.6)	238 (5.0)	1012 (10.4)	
Others	2383 (16.5)	879 (18.5)	1504 (15.5)	

a OHIS: online health information–seeking.

### Multivariate Logistic Regression Analysis of Risk Factors Associated With the Rate of Health Literacy Knowledge

The variables with statistical significance in the chi-square test (Table 1) were analyzed using multivariate logistic regression. As shown in Table 2, participants aged 46‐60 years (odds ratio [OR] 0.782, 95% CI 0.695-0.880) or more than 61 years (OR 0.298, 95% CI 0.260-0.341) were less likely to have OHIS experience than those younger than 20 years. Participants who belonged to ethnic minorities (OR 0.621, 95% CI 0.513-0.752) were less likely to have OHIS experience than Han Chinese participants. Participants from rural areas (OR 0.815, 95% CI 0.734-0.906) were less likely to have OHIS experience than urban participants. Compared to the respondents with primary school education and below, those with junior high school education (OR 2.290, 95% CI 2.000-2.622), senior high school education (OR 3.274, 95% CI 2.765-3.877), college graduate degrees (OR 5.012, 95% CI 4.163-6.033), and postgraduate education (OR 4.809, 95% CI 3.442-6.720) were more likely to have OHIS experience. Based on the participants’ occupation, compared to civil servants, teachers (OR 1.407, 95% CI 1.118-1.770) and medical staff (OR 1.359, 95% CI 1.098-1.683) were more likely to seek health information on the web, while farmers (OR 0.656, 95% CI 0.525-0.820) were less likely to seek health information on the internet.

**Table 2.** Multivariate logistic regression analysis of the factors associated with OHIS^a^ in participants.

Characteristics	OR^b^	95% CI	*P* value
**Age (years)**			
15‐45	1^c^	—^d^	—
46‐60	0.782	0.695‐0.880	<.001
61 or older	0.298	0.260‐0.341	<.001
**Gender**			
Male	1	—	—
Female	0.950	0.871‐1.036	.24
**Ethnicity**			
Han Chinese	1	—	—
Ethnic minority	0.621	0.513‐0.752	<.001
**Area of residence**			
Urban	1	—	—
Rural	0.815	0.734‐0.906	<.001
**Education**			
Primary school or less	1	—	—
Junior high school	2.290	2.000‐2.622	<.001
Senior high school	3.274	2.765‐3.877	<.001
College graduate	5.012	4.163‐6.033	<.001
Postgraduate	4.809	3.442‐6.720	<.001
**Occupation**			
Civil servants	1	—	—
Teachers	1.407	1.118‐1.770	.004
Medical staff	1.359	1.098‐1.683	.005
Staff in public institution	1.100	0.872‐1.837	.421
Students	1.279	0.979‐1.672	.072
Farmers	0.656	0.525‐0.820	<.001
Workers	0.905	0.721‐1.135	.385
Enterprise personnel	1.052	0.843‐1.313	.652
Others	1.035	0.843‐1.272	.740

a OHIS: online health information–seeking.

b OR: odds ratio.

c Reference variable.

d Not applicable.

### Health Topics Searched on the Internet by Participants With OHIS Experience

Table 3 shows the health topics searched on the internet by the study participants. Most of the participants (8483/9704, 87.4%) indicated that they sought information about health behavior and literacy. More than three-quarters of the participants used the internet to find information about infectious diseases (78.6%, 7628/9704), chronic diseases (76.0%, 7375/9704), and first aid (75.0%, 7277/9704). More than half (58.6%, 5688/9704) of the participants searched for health policy information on the web.

**Table 3.** Common health topics searched on the internet by participants with OHIS^a^ experience (N=9704).

Topics	Participants, n (%)
Health behavior and literacy	8483 (87.4)
Infectious diseases	7628 (78.6)
Chronic disease	7375 (76.0)
First aid	7277 (75.0)
Health policies	5688 (58.6)

a OHIS: online health information–seeking.

### Sources of Web-Based Health Information Among Participants With OHIS Experience

Participants reported 1 or more different sources of health information that they sought on the web. The majority of participants (7468/9704, 77.0%) used WeChat. More than half of the participants indicated that search engines (5547/9704, 57.2%) and short-video apps (5359/9704, 55.2%) were their main sources. A total of 4079 out of 9704 (42.0%) participants used Weibo. Websites and web-based courses were used for seeking health information on the web by 36.3% (3527/9704) and 31.3% (3034/9704) of participants, respectively. The prevalence of the source of web-based health information among the participants with OHIS experience is shown in Table 4.

**Table 4.** Source of online health information among the participants with OHIS experience (N=9704).

Sources	Participants, n (%)
WeChat	7468 (77.0)
Weibo	4079 (42.0)
Search engines	5547 (57.2)
Websites	3527 (36.3)
Web-based courses	3034 (31.3)
Short-video apps	5359 (55.2)
Others	418 (4.3)

a OHIS: online health information–seeking.

## Discussion

### Principal Findings

In this study, more than 67% of participants sought health information on the internet, which is similar to recent studies reporting extensive internet use and highly prevalent OHIS [22,24,26,33]. The internet has the highest usage rate among health information sources [5-7]. The results from our study suggested that among the Chongqing population, the internet has become a major source for seeking health information, which is consistent with other reports. How to provide more high-quality health information on the web may be a key objective for public health policies and practices in Chongqing.

In this study, younger participants were more likely to seek health information on the web than older participants. Similar results were also reported in some previous studies [22,30,33,34]. IT, including health IT, is usually accessible for younger generations [35]. Therefore, younger populations may more frequently seek health information on the internet.

We also found evidence of disparities in OHIS by ethnicity in Chongqing. This result was similar to those of previous studies showing that racial or ethnic minorities were less likely to use web-based resources to seek health information [33,36,37]. A possible reason may be that diﬀerences in cultural values, care preferences, and perceived beneﬁts of web-based health information likely contributed to these diﬀerential rates of use [36-38]. Previous research has shown that health communication in a multicultural society mainly takes the dominant culture into account, often neglecting those of nondominant groups [39-41]. In China, Han is the dominant ethnicity. This is likely secondary in part to the limited availability of web-based sources such as health-related websites and patient-provider portals in ethnic minorities other than the majority [42]. In Chongqing, the population of ethnic minorities is more than 2 million [32]. Considering the benefits of OHIS, providing web-based health information that is suitable for racial or ethnic minorities may contribute to health improvement in the multiethnic region.

Participants who lived in urban areas were more likely to use the internet to seek health information. This result corroborates previous findings that the urban population had a higher rate of OHIS [33,34,43,44]. A possible reason may be the digital divide between the urban and rural participants in this study. Although there are more than 1 billion netizens in China, people living in urban areas more easily access the internet [45]. Goldner et al [44] reported that lower internet access was associated with fewer web-based health behaviors. Therefore, eliminating the digital divide may help the rural population benefit from these resources.

In this study, a higher education level was associated with the highest odds of OHIS among the Chongqing population. This finding was in line with those of previous studies [22,27,29,30,33,34,38]. Seeking health information on the web requires not only access to technology but also the ability to retrieve, understand, and use information [46]. In addition, the vast majority of web-based patient resources contain health information that is above the reading level of most users [47,48]. Although a substantial proportion of lesser educated individuals have signiﬁcant health care needs, they often encounter diﬃculties in ﬁnding acceptable information on the web [49]. Continued eﬀorts to ensure that web-based health information is easy to read, understand, and retrieve are needed.

We also found that occupation was associated with the rate of OHIS behavior. In this study, participants who were medical staff or teachers were more likely to use the internet to seek health information. The reason may be that medical staff and teachers more often have higher education levels, higher socioeconomic status, and easier access to web-based resources. In addition, due to their duties, medical staff and teachers often conduct health education for patients and students, and the internet has a tremendous amount and variety of health-related information [3,4]. Therefore, they may have a higher ability and willingness to seek health information on the internet [50]. Farmers in our study were less likely to use web-based sources for seeking health information. This finding was similar to that of a previous study in Zhejiang province [29]. Farmers always live in rural areas. Due to the digital divide between urban and rural areas, farmers may have more difficulty accessing the internet [44,45].

In this study, participants reported seeking web-based health information for a broad range of health topics, including health behavior and literacy, infectious diseases, chronic disease, first aid, and health policies. Health behavior and literacy are the most common topics searched on the internet. This finding is similar to those of previous studies in which health behavior, lifestyle, and health science popularization were most commonly searched for types of web-based health information among the Chinese general or younger population [27,28]. In recent years, the Chinese government has tended to use social media to improve public health literacy and health status among Chinese citizens and has encouraged the dissemination of health science popularization information in various ways [51-53]. The increasing popularity of social media and the ever-growing number of official accounts of health science popularization might have attracted many Chinese netizens to use such information to improve their health behavior and literacy.

We found that WeChat was the most commonly used source for OHIS on the internet. A previous study reported that the number of WeChat customers has exceeded 900 million, with 150 million customers using the web for at least 2 hours every day [54]. A nationwide survey in China found that one-third of participants regularly read health information articles on WeChat, and more than 90% of the participants chose to use WeChat for health information seeking, indicating that a WeChat account is the most popular platform for acquiring health information in China [55]. However, due to growing OHIS behaviors, increasing numbers of nonauthorized social media accounts share biased or inaccurate health information; continued eﬀorts are needed to improve the quality of health information on social media [56-58]. Improving perceived eHealth literacy among netizens and feedback-seeking behavior in digital environments would be useful to increase OHIS, and may finally help to improve public health among Chongqing Population [59,60].

### Limitations

This study has several limitations. First, this study was conducted in Chongqing, and the results may not be generalizable to the general population in China. Second, due to the nature of a cross-sectional survey, it is difficult to draw causal conclusions. Third, as a hot topic of OHIS in this study, failing to examine the effect of the COVID-19 on OHIS was a significant study limitation.

### Conclusion

In summary, OHIS is prevalent in Chongqing. We found that participants who were younger, lived in an urban area, had a higher educational level, and worked as medical staff or teachers were more likely to engage in OHIS, while ethnic minorities and farmers were less likely to seek health information on the web. Among the Chongqing population, the most common topics sought on the internet were health behavior and literacy, and the most popular method of OHIS was through WeChat. According to the identified influencing factors, future research could focus on bridging the digital divide between urban and rural areas, providing higher-quality web-based health information, and examining cultural barriers to health information access among ethnic minority groups. These efforts may help to enhance Chongqing residents’ ability to obtain web-based health resources and ultimately improve public health outcomes.

## Abbreviations

OHIS: online health information–seeking
OR: odds ratio
