# Correction: Online Health Information–Seeking Behaviors Among the Chongqing Population: Cross-Sectional Questionnaire Study

**DOI:** 10.2196/77500

**Published:** 2025-05-30

**Authors:** Honghui Rong, Lu Lu, Miao He, Tian Guo, Xian Li, Qingliu Tao, Yixin Li, Chuanfen Zheng, Ling Zhang, Fengju Li, Dali Yi, Enyu Lei, Ting Luo, Qinghua Yang, Ji-an Chen

**Affiliations:** 1 Department of Health Education, School of Military Preventive Medicine, Army Medical University (Third Military Medical University), Gaotanyan Street No 30, Shapingba District, Chongqing, 400038, China, 86 02368771579; 2 Chongqing Health Education Institute, Chongqing, China

In “Online Health Information–Seeking Behaviors Among the Chongqing Population: Cross-Sectional Questionnaire Study” (JMIR Form Res 2025;9:e56028) the authors made one correction.

The affiliation of author Lu Lu, Chuanfen Zheng, Fengju Li, and Ting Luo, has been changed from affiliation 2:

Chongqing Health Education Institute

to affiliation 1:

Department of Health Education, School of Military Preventive Medicine, Army Medical University (Third Military Medical University), Chongqing, China

The correction will appear in the online version of the paper on the JMIR Publications website, together with the publication of this correction notice. Because this was made after submission to PubMed, PubMed Central, and other full-text repositories, the corrected article has also been resubmitted to those repositories.

